# Multiple sclerosis as an asynchronous neuroinflammatory system

**DOI:** 10.3389/fimmu.2026.1877094

**Published:** 2026-06-29

**Authors:** Emanuele D’Amico, Aurora Zanghì, Daniel Ontaneda

**Affiliations:** 1 Department of Medical and Surgical Sciences, University of Foggia, Foggia, Italy; 2 Mellen Center for Multiple Sclerosis Treatment and Research, Cleveland Clinic, Cleveland, OH, United States

**Keywords:** asynchronous dynamics, clinical trial modeling, disease progression, multidimensional state space, multiple sclerosis

## Abstract

Multiple sclerosis (MS) has traditionally been conceptualized through an event-based framework in which relapses, lesion accumulation, and disability progression evolve along a linear temporal axis. However, converging pathological, imaging, biomarker, and clinical trial evidence indicates that inflammatory activity, chronic lesion expansion, axonal degeneration, and network reorganization unfold with partially independent temporal dynamics. The 2024 revisions of the McDonald criteria formally incorporate markers of immune persistence and chronic inflammatory organization. Dissemination in space (DIS) remains the diagnostic entry point with a relaxation for the need of dissemination in time (DIT) yet prognosis cannot be reduced to event frequency alone. Disability accumulation reflects the interaction between Relapse-associated worsening (RAW) and progression independent of relapse activity (PIRA), revealing a decomposable hazard structure that challenges relapse-centered modeling. Traditional endpoints such as annualized relapse rate (ARR) and confirmed disability progression (CDP) capture partial projections of a higher-dimensional disease process. MS should therefore be understood not as an event-driven disorder but as a dynamically evolving system. Biological onset occurs before clinical presentation, diagnosis occurs at a threshold; progression unfolds as a trajectory through a multidimensional state space (MSS). Recognizing this distinction has direct implications for prognostic inference, trial design, biomarker integration, and state-dependent therapeutic modeling.

## 1 Introduction

Multiple sclerosis (MS) is not a temporally coherent disease. Inflammatory activity, axonal injury, cortical demyelination, network reorganization, and clinical disability do not evolve in parallel, nor do they share a common temporal scale (1, 2). Relapses may subside while structural degeneration continues. Brain atrophy may progress during apparent clinical stability (3, 4). Chronic active lesions may expand without discrete clinical events (5–7). Disability may accumulate in the absence of overt inflammatory events (3). The system is intrinsically asynchronous. Importantly, biological asynchrony should not be interpreted as biological discontinuity. Inflammatory and degenerative processes likely fluctuate continuously over time, waxing and waning across different lesions, central nervous system central nervous system (CNS) compartments, and functional systems, even during periods of apparent clinical stability.

This asynchrony challenges a long-standing clinical belief: that inflammatory episodes and structural damage are tightly coupled (8). Historically, relapse frequency, relapse severity, and degree of recovery were treated as proxies for cumulative tissue injury. Event-based metrics—annualized relapse rate (ARR), confirmed disability progression (CDP), lesion accrual—became the operational language through which disease activity and treatment efficacy were quantified (9). These measures remain clinically useful. Yet accumulating evidence indicates that they capture only part of the underlying biological process.

Pathological and imaging studies reveal sustained microglial activation at lesion borders, compartmentalized immune aggregates within the meninges, and diffuse axonal transection extending beyond focal inflammatory lesions in the white matter (2, 8, 10). Quantitative magnetic resonance imaging suggested that deep grey matter loss and cortical thinning proceed in a largely continuous fashion (11–13). Biomarkers of neuroaxonal injury fluctuate independently of clinical relapse (10). The resulting picture is not one of episodic propagation, but of interacting processes evolving with partially independent dynamics.

The 2024 revisions of the diagnostic criteria focus on diagnosis, and understandably do not attempt to fully describe the biological complexity of the evolution of MS (14, 15). The criteria do acknowledge the biological underpinning of MS by incorporating markers such as intrathecal immunoglobulin synthesis, cortical lesions, the central vein sign, and paramagnetic rim lesions. The diagnostic framework increasingly acknowledges persistence, anatomic lesion location, and chronic inflammatory microenvironments. Diagnosis remains anchored in dissemination in space (DIS) with a greater relaxation regarding the demonstration of dissemination in time Time (DIT), yet the biology admitted within those axes extends beyond simple recurrence. The criteria also, importantly, acknowledge that the biological onset of MS occurs prior to the clinical onset and allows for a diagnosis of MS even in those without symptoms.

The biological onset of MS likely precedes both clinical manifestation and radiological recognition by years. Current evidence supports a multifactorial and probabilistic model in which genetic susceptibility, infectious exposures, environmental influences, and immune dysregulation interact stochastically over time. The emerging concept of an MS prodrome further supports the existence of a prolonged preclinical phase characterized by subtle systemic and neurological alterations before the diagnostic threshold is crossed.

These concepts raise a broader question that extends beyond classification. If MS evolves through asynchronous and interacting processes, how should activity, progression, prognosis, and therapeutic response be conceptualized once the diagnostic threshold has been crossed?

In this Perspective, we argue that DIS and DIT can define entry into disease, but prognosis reflects the geometry of subsequent trajectories within a multidimensional biological state space. Recognizing the distinction between threshold and trajectory reframes how populations are defined, how outcomes are interpreted, and how disease evolution is modeled in the post-2024 era.

## 2 The event-based model and its structural limits

For decades, MS has been conceptualized through an event-based framework, in which discrete pathological episodes causing focal tissue injury—whether clinically manifest or asymptomatic—may be associated with relapses, while cumulative disability reflects the additive consequences of incomplete recovery from such events (16). Disease evolution is thus interpreted as a sequence of temporally separated insults along a linear timeline (17). Statistical models followed naturally from this intuition: relapse frequency quantified inflammatory intensity; time to CDP captured structural decline; lesion accumulation indexed disease propagation (18).

This architecture rests on an implicit assumption of temporal coupling. Inflammatory activity, structural damage, and clinical disability are presumed to evolve synchronously, such that event frequency approximates cumulative biological burden (19). The success of relapse-reducing therapies in the early disease phase reinforced this view, as reductions in clinical and radiological activity translated into measurable short-term benefits (9).

However, the event-based model is structurally limited. Longitudinal data suggest that relapse burden explains only a portion of the variance in long-term disability trajectories (20). Some individuals with frequent early relapses remain stable over extended periods, whereas others with modest inflammatory activity accumulate progressive impairment (21). The phenomenon of progression independent of relapse activity (PIRA) makes explicit that structural decline may proceed without temporally proximate inflammatory peaks (4, 22, 23). Conversely, substantial inflammatory activity may occur without proportional disability accrual (24).

The limitation is not empirical inconsistency but model incompleteness. Event-based representations treat observed episodes as sufficient summaries of disease intensity. Yet if inflammatory persistence, chronic lesion expansion, and neurodegenerative processes evolve on partially independent time scales, event counts cannot capture the full state of the system (25, 26). Disability progression becomes not merely the accumulation of past events but the manifestation of ongoing processes that may be only partially observed (25–28).

Thus, the central issue is not that the event-based model is incorrect. It is that it presupposes synchronicity within a biologically asynchronous system (29, 30). When processes operate on different clocks, linear accumulation along a single temporal axis becomes an approximation rather than a structural description (31).

## 3 The 2024 criteria as a revealing moment

The 2024 revisions of the McDonald criteria while not redefining the biological nature of MS, clarifies it (32–35). By incorporating markers such as intrathecal immunoglobulin synthesis, the central vein sign, and paramagnetic rim lesions, the diagnostic framework formally acknowledges processes that are temporally extended and structurally organized (33, 36–40). Immune persistence may substitute for radiological recurrence; lesion identity increasingly reflects venocentric architecture and chronic inflammatory microenvironments rather than simple anatomical multiplicity (41).

This recalibration is diagnostically motivated—improving specificity and reducing misclassification—but its conceptual implications extend further. The criteria now admit, within a formally spatial–temporal structure, evidence of sustained inflammatory circuits and slowly expanding lesions that may evolve independently of overt clinical events. In doing so, they make explicit what longitudinal pathology and imaging have long suggested: that recurrence is not synonymous with activity, and that absence of relapse does not imply biological quiescence. Furthermore, the criteria now recognizes that the biological processes in MS may occur prior to clinical manifestations of the disease an allowing a diagnosis, as long as there is diagnostic certainty.

Importantly, the revision does not abandon DIS and DIT. Instead, it alters the informational density of those axes. Time is no longer as an important feature for confirming the diagnosis of MS by substituting interval-based recurrence with the presence of specific biological markers. DIS is no longer merely geographic distribution but may encode lesion topology and microenvironmental stability. The structure remains threshold-based. The biology admitted within it becomes richer and less episodic.

The consequence is epistemological rather than procedural. Once diagnosis may be established in the presence of markers of sustained inflammation or chronic lesion activity, the traditional equivalence between event frequency and disease intensity becomes further destabilized. Patients may enter the disease category with biological configurations that already reflect partially decoupled inflammatory and degenerative dynamics. The diagnostic boundary, therefore, no longer implicitly supports a strictly event-driven model of evolution.

In this sense, the 2024 revision functions as a revealing moment. It exposes the limitations of linear, relapse-centered representations not by invalidating them, but by highlighting their incompleteness. The question that follows is no longer how to diagnose disease, but how to represent its subsequent trajectory once asynchronous processes are formally acknowledged at entry.

## 4 From diagnosis to prognosis: the problem of trajectory

If MS is biologically asynchronous, and if the 2024 revision formally acknowledges processes that extend beyond episodic recurrence, then diagnosis and prognosis must be conceptually disentangled. DIS/DIT establishes that a pathological process consistent with MS is present. It does not specify how that process will evolve.

Prognosis has historically been inferred from early inflammatory metrics: relapse frequency, lesion burden, and recovery kinetics (42–44). These variables were assumed to approximate the intensity of the underlying disease and, by extension, its future course. Yet if inflammatory persistence, chronic lesion expansion, structural degeneration, and network reorganization operate on partially independent time scales, then early event frequency cannot fully determine long-term trajectory (20, 45–47).

Two individuals may satisfy identical diagnostic thresholds and exhibit comparable early relapse profiles, yet diverge substantially in subsequent disability accumulation (48). Such divergence reflects differences not only in inflammatory amplitude but in structural reserve, repair capacity, compartmentalized immune activity, and network adaptability (47, 49, 50). The relevant prognostic question is therefore not whether dissemination has occurred, but how the interacting components of the disease system evolve over time.

This shift reframes disease progression from a sequence of events to a trajectory through a Multidimensional State Space (MSS) (51). Clinical milestones—relapse, new lesion, CDP—become observable markers along a path whose geometry is not fully captured by their frequency alone. Velocity, acceleration, regime stability, and susceptibility to perturbation emerge as more informative descriptors of evolution than simple event counts (17, 51, 52).

The challenge that follows is methodological. If disease unfolds as a trajectory shaped by interacting and asynchronous processes, then statistical models must represent that structure explicitly rather than infer it indirectly from episodic observations (53).

The diagnostic threshold defines disease entry, whereas prognosis reflects the subsequent organization of biological trajectories.

From a clinical perspective, this framework may help explain why patients with apparently similar inflammatory burden can experience profoundly different long-term outcomes.

## 5 Modeling disease evolution: from clinical data to latent dynamics

The shift from event-based reasoning to trajectory-based reasoning is not speculative. MS research has already begun to move beyond simple event counting, even if this evolution remains methodologically fragmented.

CDP models traditionally relied on negative binomial models for relapse counts and Cox proportional hazards models (19, 54). These frameworks remain regulatory standards, yet they operationalize disease activity through event-defined endpoints. They describe observable manifestations, not the structural organization of the evolving system.

Evidence from large real-world registries—including MSBase—highlighted that relapse burden explains only a fraction of long-term disability variance (55, 56). Linear mixed-effects modeling of Expanded Disability Status Scale trajectories reveals pronounced inter-individual heterogeneity in progression slopes, often independent of baseline inflammatory intensity (57, 58). Disability evolution does not fluctuate around a single population average; it organizes into differentiated patterns.

Trajectory clustering approaches make this structure explicit. Growth mixture modeling and latent class analyses identify distinct evolution profiles—rapid accumulators, stable-low trajectories, delayed accelerators—despite comparable early inflammatory metrics (47, 52, 59). These findings indicate that progression reflects differentiated dynamic regimes rather than stochastic perturbations of a homogeneous slope.

Joint longitudinal–survival models extend this perspective by integrating evolving biomarkers such as serum neurofilament light chain into time-to-disability analyses (57). In these models, progression risk becomes conditional on the current biological configuration rather than fixed baseline characteristics. Hidden Markov models applied to MS natural history data further develop this logic by modeling probabilistic transitions between latent disease regimes—for example, inflammation-dominant and progression-dominant phases—thereby abandoning the assumption of temporally uniform risk (60–62). Clinical milestones emerge as observable projections of underlying state transitions rather than isolated drivers of change.

Taken together, these analytic approaches support a structural reinterpretation of MS evolution as a partially observed, state-dependent process whose organization cannot be reduced to event frequency alone.

## 6 Systems biology and dynamic phenotyping in clinical MS

If progression organizes into differentiated dynamic regimes, its biological substrate must be correspondingly multidimensional. MS reflects the coupled interaction of immune activation, compartmentalized CNS inflammation, axonal degeneration, glial reactivity, vascular dysfunction, and network reorganization operating across distinct temporal scales (16, 63). Peripheral inflammatory bursts, chronic lesion expansion, mitochondrial impairment, synaptic remodeling, and compensatory network plasticity unfold without temporal coherence (64, 65). Their interaction generates evolving configurations that may stabilize, reorganize, or destabilize over time (66). These processes likely remain biologically active in a continuous manner, although with fluctuating intensity across time and anatomical compartments. Acute relapses may therefore represent transient amplifications superimposed on a persistent inflammatory and degenerative background rather than isolated pathological events.

Representation-based and machine learning approaches provide empirical access to this multidimensional organization (16, 67). Unsupervised analyses of longitudinal imaging, biomarker, and clinical data identify structurally distinct trajectories despite similar relapse histories (68, 69). Dimensionality reduction techniques reveal latent axes of variation not captured by conventional endpoints, situating patients within a continuously evolving biological landscape.

Monitoring, in this context, becomes the interpretation of configuration change. Biomarkers such as sNfL, quantitative measures of deep grey matter volume, and slowly expanding lesion dynamics function as partial readouts of an evolving system whose internal organization may shift even in the absence of overt clinical events (66, 67, 70, 71).

## 7 Trial populations, PIRA, and multidimensional treatment effects

The implications of this structural perspective become most evident in therapeutic evaluation. Disability accumulation reflects at least two partially separable processes: relapse associated worsening-RAW and PIRA. Trials and pooled datasets demonstrate that a substantial proportion of CDP events occur without temporally proximate relapse, undermining the assumption that relapse suppression alone prevents long-term worsening (72).

ARR and CDP remain indispensable endpoints, yet both compress a multidimensional biological evolution into scalar summaries. Relapse counts abstract away severity and structural consequence; discrete disability thresholds fragment what is in reality a continuous trajectory. As high-efficacy therapies attenuate inflammatory amplitude, slower structural mechanisms—including chronic lesion expansion, axonal degeneration, and compartmentalized inflammation—become increasingly salient determinants of long-term outcome. Therapeutic effects therefore distribute unevenly across biological dimensions. Anti-inflammatory disease modifying medications predominantly modulate inflammatory fluctuations, whereas neuroprotective or reparative strategies may influence structural vulnerability without proportionally altering relapse frequency. Interpreting efficacy requires analytic frameworks capable of distinguishing relapse-linked from relapse-independent progression and of integrating longitudinal biological signals into outcome assessment.

A multidimensional view of MS also carries important therapeutic implications. Suppression of acute inflammatory activity remains essential, particularly during the earlier phases of disease, but increasingly appears insufficient to fully modify long-term disease trajectories once compartmentalized inflammation, chronic lesion expansion, and neuroaxonal degeneration become established. In this framework, therapeutic efficacy may depend not only on treatment potency, but also on the biological state in which intervention occurs.

Early disease phases may be dominated by peripheral inflammatory fluctuations that are highly responsive to anti-inflammatory therapies, whereas later phases may increasingly reflect smouldering inflammation, failure of tissue repair, and progressive loss of biological reserve.

Therapeutic strategies may therefore need to evolve from uniform relapse-centered escalation models toward state-dependent approaches integrating anti-inflammatory, neuroprotective, remyelinating, and network-preserving interventions according to the dominant biological processes active at different stages of disease evolution.

Such a perspective may also help explain why substantial suppression of focal inflammatory activity does not always translate into complete prevention of long-term disability accumulation and supports the development of therapeutic strategies tailored not only to disease stage, but also to the dominant biological processes driving individual disease trajectories.

MS is diagnosed at a threshold, however biologically it evolves as a trajectory governed by interacting and asynchronous processes. Trial methodology must align with this structural organization if it aims to capture meaningful modification of disease course.

## 8 Conclusion

MS is not an event-driven disease intermittently interrupted by relapses, but a continuously evolving biological system in which inflammatory and degenerative processes fluctuate across partially independent temporal scales. Inflammatory bursts, structural degeneration, and network reorganization unfold on different clocks, generating trajectories that cannot be fully captured by event-based representations.Relapses and disability milestones are not the engine of progression; they are its visible contours. Much of the pathological substrate likely persists as a smouldering and continuously fluctuating process beneath overt clinical manifestations. The deeper architecture of disease lies in the interaction between immune persistence, lesion chronicity, structural reserve, and adaptive capacity. The distinction between RAW and PIRA makes explicit what has long been implicit: progression can advance even in the absence of overt inflammatory peaks.

Prognosis, therefore, cannot be reduced to event frequency. It reflects the geometry of a trajectory moving through a multidimensional biological landscape. Statistical models and trial endpoints that ignore this structure risk mistaking surface fluctuation for structural modification.

**Figure 1:**
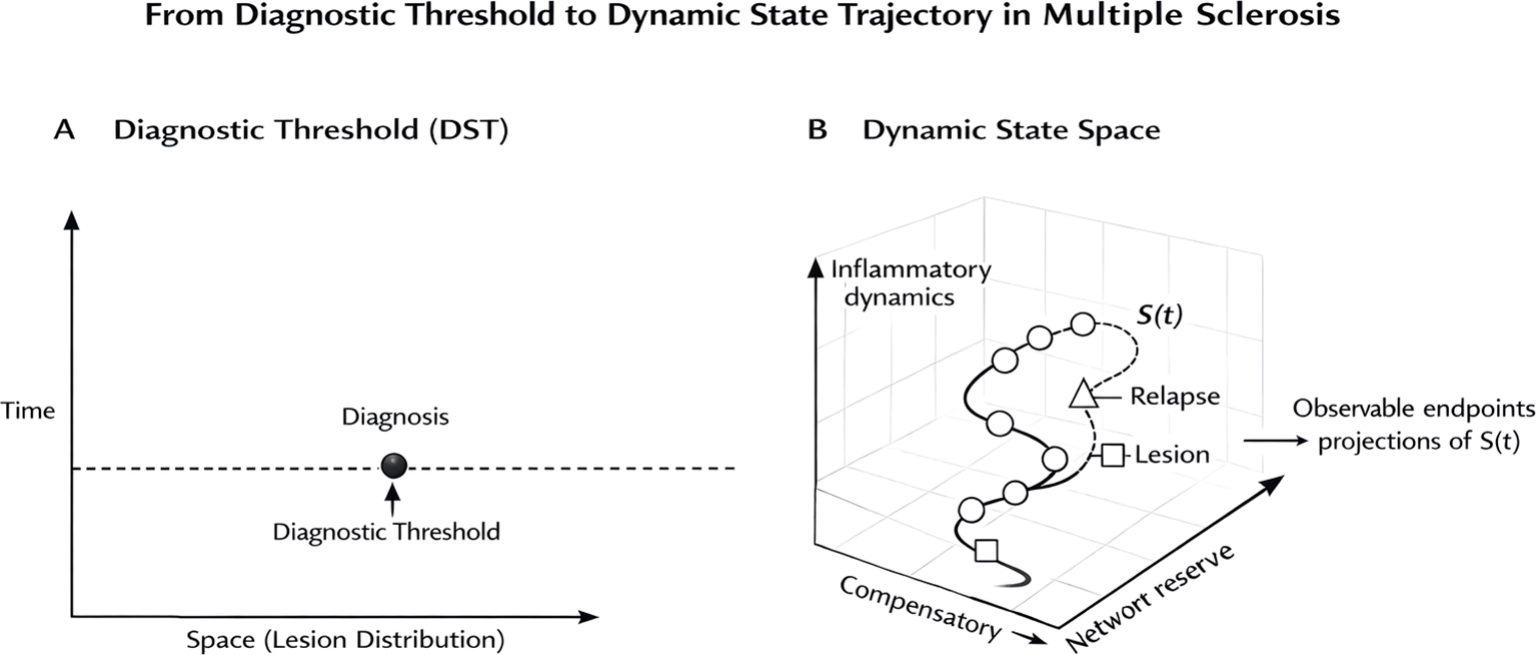
From diagnostic threshold to dynamic state trajectory in multiple sclerosis. **(A)** Diagnosis is established when the spatial–temporal boundary defined by Dissemination in Space and Time (DST) is crossed. This threshold marks disease entry but does not define future evolution. **(B)** Multiple sclerosis unfolds as a trajectory S(t) within a multidimensional biological state space composed of interacting and partially asynchronous processes. Clinical events and disability milestones represent observable projections of the evolving state rather than discrete causal drivers of progression.

MS is diagnosed at a moment in time, but its biology evolves through changing configurations and is ultimately expressed as a clinical trajectory (**Figure 1**).

Understanding that distinction is not semantic—it is the difference between counting events and comprehending disease.

## Data Availability

The original contributions presented in the study are included in the article/supplementary material. Further inquiries can be directed to the corresponding author.

## References

[1] Trapp BD Nave KA . Multiple sclerosis: an immune or neurodegenerative disorder? Annu Rev Neurosci. (2008) 31:247–69. doi:top 10.1146/annurev.neuro.30.051606.094313 18558855

[2] Lassmann H . Multiple sclerosis pathology. Cold Spring Harb Perspect Med. (2018) 8. doi:top 10.1101/cshperspect.a028936 29358320 PMC5830904

[3] Bsteh G Marti S Hammer H Krajnc N Guger M Di Pauli F . Dissecting definitions of disability accrual in relapsing multiple sclerosis-have we reached standardization yet? Mult Scler. (2026) 32:179–91. doi:top 10.1177/13524585251396283 41351456 PMC12916871

[4] Kappos L Wolinsky JS Giovannoni G Arnold DL Wang Q Bernasconi C . Contribution of relapse-independent progression vs relapse-associated worsening to overall confirmed disability accumulation in typical relapsing multiple sclerosis in a pooled analysis of 2 randomized clinical trials. JAMA Neurol. (2020) 77:1132–40. doi:top 10.1001/jamaneurol.2020.1568 32511687 PMC7281382

[5] Absinta M Sati P Masuzzo F Nair G Sethi V Kolb H . Association of chronic active multiple sclerosis lesions with disability *in vivo*. JAMA Neurol. (2019) 76:1474–83. doi:top 10.1001/jamaneurol.2019.2399 31403674 PMC6692692

[6] Polvinen E Matilainen M Nylund M Sucksdorff M Airas LM . TSPO-detectable chronic active lesions predict disease progression in multiple sclerosis. Neurol Neuroimmunol Neuroinflamm. (2023) 10. doi:top 10.1212/nxi.0000000000200133 37349108 PMC10291892

[7] Preziosa P Schoonheim MM . Ongoing axonal injury in chronic active lesions in multiple sclerosis: *in vivo* quantification using serum neurofilament. Neurology. (2021) 97:257–8. doi:top 10.1212/wnl.0000000000012331 34088879

[8] Wafa M Giovannoni G . Lost in space, lost in time: a clinician's proposed approach to the diagnosis of multiple sclerosis. Mult Scler Relat Disord. (2026) 107:106989. doi:top 10.1016/j.msard.2026.106989 41579715

[9] Montobbio N Cordioli C Signori A Bovis F Capra R Sormani MP . Relapse-associated and relapse-independent contribution to overall expanded disability status scale progression in multiple sclerosis patients diagnosed in different eras. Ann Neurol. (2024) 97:95–103. doi:top 10.1002/ana.27093 39381962 PMC11683181

[10] Andravizou A Stavropoulou De Lorenzo S Kesidou E Michailidou I Parissis D Boziki MK . The time trajectory of choroid plexus enlargement in multiple sclerosis. Healthcare (Basel). (2024) 12. doi:top 10.3390/healthcare12070768 38610190 PMC11011748

[11] Maiworm M Hamid C Wagner M Nöth U Deichmann R Seiler A . Multiparametric quantitative MRI reveals progressive cortical damage over time in clinically stable relapsing-remitting MS. J Neurol Neurosurg Psychiatry. (2023) 94:786–91. doi:top 10.1136/jnnp-2022-330894 37169544

[12] Mistry N Hobart J Rog D Muhlert N Mathews J Baker D . Reconciling lesions, relapses and smouldering associated worsening: a unifying model for multiple sclerosis pathogenesis. Mult Scler Relat Disord. (2024) 88:105706. doi:top 10.1016/j.msard.2024.105706 38880031

[13] Lommers E Simon J Reuter G Delrue G Dive D Degueldre C . Multiparameter MRI quantification of microstructural tissue alterations in multiple sclerosis. NeuroImage Clin. (2019) 23:101879. doi:top 10.1016/j.nicl.2019.101879 31176293 PMC6555891

[14] Tena-Cucala R Naval-Baudin P Martínez-Yélamos A León I Muñoz-Vendrell A Bau L . The 2024 McDonald criteria show comparable sensitivity to previous criteria for late-onset multiple sclerosis. Mult Scler Relat Disord. (2025) 103:106658. doi:top 10.1016/j.msard.2025.106658 40752117

[15] Brownlee WJ Maccarrone D Nistri R Yam C Wilson H Wright S . Performance of the 2024 McDonald criteria in patients under evaluation for suspected multiple sclerosis. Neurology. (2026) 106:e214688. doi:top 10.1212/wnl.0000000000214688 41687049 PMC12938227

[16] Ganjgahi H Häring DA Aarden P Graham G Sun Y Gardiner S . AI-driven reclassification of multiple sclerosis progression. Nat Med. (2025) 31:3414–24. doi:top 10.1038/s41591-025-03901-6 40835969 PMC12532606

[17] Meier DS Weiner HL Guttmann CR . Time-series modeling of multiple sclerosis disease activity: a promising window on disease progression and repair potential? Neurotherapeutics. (2007) 4:485–98. doi:top 10.1016/j.nurt.2007.05.008 17599713 PMC7479736

[18] Mandel M Gauthier SA Guttmann CR Weiner HL Betensky RA . Estimating time to event from longitudinal categorical data: an analysis of multiple sclerosis progression. J Am Stat Assoc. (2007) 102:1254–66. doi:top 10.1007/978-3-531-92073-3_6 19081806 PMC2600443

[19] Lublin FD Häring DA Ganjgahi H Ocampo A Hatami F Čuklina J . How patients with multiple sclerosis acquire disability. Brain. (2022) 145:3147–61. doi:top 10.1093/brain/awac016 35104840 PMC9536294

[20] Scalfari A Neuhaus A Degenhardt A Rice GP Muraro PA Daumer M . The natural history of multiple sclerosis: a geographically based study 10: relapses and long-term disability. Brain. (2010) 133:1914–29. doi:top 10.1093/brain/awq118 20534650 PMC2892939

[21] Portaccio E Bellinvia A Fonderico M Pastò L Razzolini L Totaro R . Progression is independent of relapse activity in early multiple sclerosis: a real-life cohort study. Brain. (2022) 145:2796–805. doi:top 10.1093/brain/awac111 35325059

[22] Alonso R Casas M Rojas JI Eizaguirre MB Lazaro L Pita C . Progression independent of relapse activity (PIRA) in the era of high-efficacy treatments. Mult Scler Relat Disord. (2025) 104:106775. doi:top 10.1016/j.msard.2025.106775 41005018

[23] Portaccio E Betti M De Meo E Addazio I Pastò L Razzolini L . Progression independent of relapse activity in relapsing multiple sclerosis: impact and relationship with secondary progression. J Neurol. (2024) 271:5074–82. doi:top 10.1007/s00415-024-12448-4 38805052 PMC11319422

[24] Maarouf A Stellmann JP Rico A Boutiere C Demortiere S Durozard P . Active and non-active progression independent of relapse activity within the first 20 years of relapsing multiple sclerosis. J Neurol Neurosurg Psychiatry. (2024) 95:974–8. doi:top 10.1136/jnnp-2024-333597 38719433

[25] Cagol A Schaedelin S Barakovic M Benkert P Todea RA Rahmanzadeh R . Association of brain atrophy with disease progression independent of relapse activity in patients with relapsing multiple sclerosis. JAMA Neurol. (2022) 79:682–92. doi:top 10.1001/jamaneurol.2022.1025 35575778 PMC9112138

[26] Calabrese M Preziosa P Scalfari A Colato E Marastoni D Absinta M . Determinants and biomarkers of progression independent of relapses in multiple sclerosis. Ann Neurol. (2024) 96:1–20. doi:top 10.1002/ana.26913 38568026

[27] Tur C Carbonell-Mirabent P Cobo-Calvo ÁChecktae Otero-Romero S Arrambide G Midaglia L . Association of early progression independent of relapse activity with long-term disability after a first demyelinating event in multiple sclerosis. JAMA Neurol. (2023) 80:151–60. doi:top 10.1001/jamaneurol.2022.4655 36534392 PMC9856884

[28] Zárate MA Marrodan M Piedrabuena MA Fiol MP Ysrraelit MC Correale J . Development of early progression independent of relapse activity significantly impacts on disability accumulation in patients with multiple sclerosis. Mult Scler Relat Disord. (2025) 100:106529. 40398063 10.1016/j.msard.2025.106529

[29] Ciccarelli O Barkhof F Calabrese M De Stefano N Eshaghi A Filippi M . Using the progression independent of relapse activity framework to unveil the pathobiological foundations of multiple sclerosis. Neurology. (2024) 103:e209444. doi:top 10.1212/wnl.0000000000209444 38889384 PMC11226318

[30] Simone M Lucisano G Guerra T Paolicelli D Rocca MA Brescia Morra V . Disability trajectories by progression independent of relapse activity status differ in pediatric, adult and late-onset multiple sclerosis. J Neurol. (2024) 271:6782–90. doi:top 10.1007/s00415-024-12638-0 39179712 PMC11447039

[31] Iaffaldano P Portaccio E Lucisano G Simone M Manni A Guerra T . Multiple sclerosis progression and relapse activity in children. JAMA Neurol. (2024) 81:50–8. doi:top 10.1001/jamaneurol.2023.4455 38010712 PMC10682937

[32] Montalban X Lebrun-Frénay C Oh J Arrambide G Moccia M Pia Amato M . Diagnosis of multiple sclerosis: 2024 revisions of the McDonald criteria. Lancet Neurol. (2025) 24:850–65. doi:top 10.1016/s1474-4422(25)00270-4 40975101

[33] Barkhof F Reich DS Oh J Rocca MA Li DKB Sati P . 2024 MAGNIMS-CMSC-NAIMS consensus recommendations on the use of MRI for the diagnosis of multiple sclerosis. Lancet Neurol. (2025) 24:866–79. doi:top 10.1016/s1474-4422(25)00304-7 40975102

[34] Manazoğlu H Kürtüncü M . Redefining multiple sclerosis: 2024 McDonald diagnostic criteria. Turkish J Neurol. (2025) 31:255–69.

[35] Samara A Ontaneda D . Evolving the diagnosis of multiple sclerosis: a new landscape in light of the 2024 McDonald criteria. Biomedicines. (2025) 13. doi:top 10.3390/biomedicines13112590 41301685 PMC12650676

[36] Agrawal M Sridhar S Surendran AK Bhatt A Sriwastava S . Diagnostic evolution in multiple sclerosis: a narrative review of the McDonald criteria from 2001 to 2024. Mult Scler Relat Disord. (2025) 104:106756. doi:top 10.1016/j.msard.2025.106756 41101247

[37] Konzen VR Finkelsztejn A Dos Santos RP de Souza AM Ruffini ML Londero RG . The central vein sign and paramagnetic rim lesions: biomarkers for an accurate differential diagnosis between multiple sclerosis and migraine. Radiol Bras. (2025) 58:e20250015. doi:top 10.1590/0100-3984.2025.0015 41036299 PMC12482283

[38] Levraut M Landes-Chateau C Mondot L Cohen M Lebrun-Frenay C . The kappa free light chains index and central vein sign: two new biomarkers for multiple sclerosis diagnosis. Neurol Ther. (2025) 14:711–31. doi:top 10.1007/s40120-025-00737-7 40189723 PMC12089642

[39] Wynen M Vanden Bulcke C Borrelli S Gordaliza PM Stölting A Guisset F . Machine learning-based combination of the central vein sign, cortical lesions and paramagnetic rim lesions: a web-based tool for the diagnosis of multiple sclerosis. Brain Commun. (2026) 8:fcag079. doi:top 10.2139/ssrn.5192817 41884595 PMC13010066

[40] Rai P Bathla G Chan VEY Bagnato F Vattoth S Deng F . The 2024 update to the McDonald criteria for multiple sclerosis diagnosis: a guide for radiologists. AJR Am J Roentgenol. (2026) 226:e2533997. doi:top 10.2214/ajr.25.33997 41439773

[41] Quinn MP Kremenchutzky M Menon RS . Venocentric lesions: an MRI marker of MS? Front Neurol. (2013) 4:98. doi:top 10.3389/fneur.2013.00098 23885252 PMC3717618

[42] Binquet C Quantin C Le Teuff G Pagliano JF Abrahamowicz M Moreau T . The prognostic value of initial relapses on the evolution of disability in patients with relapsing-remitting multiple sclerosis. Neuroepidemiology. (2006) 27:45–54. doi:top 10.1159/000094380 16825794

[43] Confavreux C Vukusic S Adeleine P . Early clinical predictors and progression of irreversible disability in multiple sclerosis: an amnesic process. Brain. (2003) 126:770–82. doi:top 10.1093/brain/awg081 12615637

[44] Zanghì A Galgani S Bellantonio P Zaffaroni M Borriello G Inglese M . Relapse-associated worsening in a real-life multiple sclerosis cohort: the role of age and pyramidal phenotype. Eur J Neurol. (2023) 30:2736–44. 10.1111/ene.15910 37294976

[45] Klistorner S Barnett M Parratt J Yiannikas C Wang C Wang D . Evolution of chronic lesion tissue in relapsing-remitting patients with multiple sclerosis: an association with disease progression. Neurol Neuroimmunology Neuroinflamm. (2025) 12:e200377. doi:top 10.1212/nxi.0000000000200377 40020214 PMC11908449

[46] Scalfari A Neuhaus A Daumer M Deluca GC Muraro PA Ebers GC . Early relapses, onset of progression, and late outcome in multiple sclerosis. JAMA Neurol. (2013) 70:214–22. doi:top 10.1001/jamaneurol.2013.599 23407713

[47] Signori A Lorscheider J Vukusic S Trojano M Iaffaldano P Hillert J . Heterogeneity on long-term disability trajectories in patients with secondary progressive MS: a latent class analysis from Big MS Data network. J Neurol Neurosurg Psychiatry. (2023) 94:23–30. doi:top 10.1136/jnnp-2022-329987 36171104

[48] Krieger S Cook K Hersh CM . Understanding multiple sclerosis as a disease spectrum: above and below the clinical threshold. Curr Opin Neurol. (2024) 37:189–201. doi:top 10.1097/wco.0000000000001262 38535979 PMC11064902

[49] Vollmer TL Nair KV Williams IM Alvarez E . Multiple sclerosis phenotypes as a continuum: the role of neurologic reserve. Neurol Clin Pract. (2021) 11:342–51. doi:top 10.1038/npg.els.0000192 34476126 PMC8382415

[50] Ocampo A Hatami F Čuklina J Graham G Ganjgahi H Sun Y . Prognostic factors for worsening and improvement in multiple sclerosis using a multistate model. Mult Scler. (2024) 30:1455–67. doi:top 10.1177/13524585241275471 39340359

[51] Durso-Finley J Barile B Falet J-P Arnold D Pawlowski N Arbel T . Probabilistic temporal prediction of continuous disease trajectories and treatment effects using neural SDEs. (2024).

[52] Menezes FTL Lopes AB Alencar JMD Bichuetti DB Souza NA Cogo-Moreira H . A mixture model for differentiating longitudinal courses of multiple sclerosis. Mult Scler Relat Disord. (2024) 81:105346. doi:top 10.1016/j.msard.2023.105346 38091806

[53] Healy BC Engler D . Modeling disease-state transition heterogeneity through Bayesian variable selection. Stat Med. (2009) 28:1353–68. doi:top 10.1002/sim.3545 19206077

[54] Butzkueven H Spelman T Horakova D Hughes S Solaro C Izquierdo G . Risk of requiring a wheelchair in primary progressive multiple sclerosis: Data from the ORATORIO trial and the MSBase registry. Eur J Neurol. (2022) 29:1082–90. doi:top 10.1111/ene.14824 33724638 PMC9292576

[55] Kalincik T Butzkueven H . The MSBase registry: Informing clinical practice. Mult Scler. (2019) 25:1828–34. doi:top 10.1177/1352458519848965 31120376

[56] Stefan B Eleni K Philip VH Arnfin B Jelena S Aksel S . Accuracy of MSBase criteria to diagnose secondary progressive multiple sclerosis in large German real-world patient cohort. Mult Scler Relat Disord. (2024) 90:105844. doi:top 10.1016/j.msard.2024.105844 39197353

[57] Pellegrini F Copetti M Sormani MP Bovis F de Moor C Debray TP . Predicting disability progression in multiple sclerosis: Insights from advanced statistical modeling. Mult Scler. (2020) 26:1828–36. doi:top 10.1177/1352458519887343 31686590

[58] Guo J Olsson T Alfredsson L Hedström AK . Disability progression in multiple sclerosis: a latent class analysis of predictors. J Neurol. (2026) 273. doi:top 10.1007/s00415-026-13704-5 41721025 PMC12923449

[59] Signori A Izquierdo G Lugaresi A Hupperts R Grand'Maison F Sola P . Long-term disability trajectories in primary progressive MS patients: A latent class growth analysis. Mult Scler. (2018) 24:642–52. doi:top 10.1177/1352458517703800 28382837

[60] Kempf G Ng HS Petkau J Tremlett H Zhu F Zhao Y . Multistate modeling in long-term follow-up studies, with application to a multiple sclerosis cohort. J Clin Epidemiol. (2025) 183:111810. doi:top 10.1016/j.jclinepi.2025.111810 40300675

[61] Kotelnikova E Kiani NA Abad E Martinez-Lapiscina EH Andorra M Zubizarreta I . Dynamics and heterogeneity of brain damage in multiple sclerosis. PloS Comput Biol. (2017) 13:e1005757. doi:top 10.1371/journal.pcbi.1005757 29073203 PMC5657613

[62] Ma Y Chen H Kang J Guo X Sun C Xu J . The hidden Markov model and its applications in bioinformatics analysis. Genes Dis. (2026) 13:101729. doi:top 10.1016/j.gendis.2025.101729 41069576 PMC12505677

[63] Bittner S Zipp F . Progression in multiple sclerosis - a long-term problem. Curr Opin Neurol. (2022) 35:293–8. doi:top 10.1016/s0140-6736(23)01473-3 35674071

[64] Groppa S Gonzalez-Escamilla G Eshaghi A Meuth SG Ciccarelli O . Linking immune-mediated damage to neurodegeneration in multiple sclerosis: could network-based MRI help? Brain Commun. (2021) 3:fcab237. doi:top 10.1093/braincomms/fcab237 34729480 PMC8557667

[65] Maier S Barcutean L Andone S Manu D Sarmasan E Bajko Z . Recent progress in the identification of early transition biomarkers from relapsing-remitting to progressive multiple sclerosis. Int J Mol Sci. (2023) 24. doi:top 10.3390/ijms24054375 36901807 PMC10002756

[66] Eshaghi A Young AL Wijeratne PA Prados F Arnold DL Narayanan S . Identifying multiple sclerosis subtypes using unsupervised machine learning and MRI data. Nat Commun. (2021) 12:2078. doi:top 10.1038/s41467-021-22265-2 33824310 PMC8024377

[67] Campanioni S Veiga C Prieto-González JM González-Nóvoa JA Busto L Martinez C . Explainable machine learning on baseline MRI predicts multiple sclerosis trajectory descriptors. PloS One. (2024) 19:e0306999. doi:top 10.1371/journal.pone.0306999 39012871 PMC11251627

[68] Andorra M Freire A Zubizarreta I de Rosbo NK Bos SD Rinas M . Predicting disease severity in multiple sclerosis using multimodal data and machine learning. J Neurol. (2024) 271:1133–49. doi:top 10.1007/s00415-023-12132-z 38133801 PMC10896787

[69] Gross C Schulte-Mecklenbeck A Steinberg O Wirth T Lauks S Bittner S . Multiple sclerosis endophenotypes identified by high-dimensional blood signatures are associated with distinct disease trajectories. Sci Transl Med. (2024) 16:eade8560. doi:top 10.1126/scitranslmed.ade8560 38536936

[70] Bagnato F Sati P Hemond CC Elliott C Gauthier SA Harrison DM . Imaging chronic active lesions in multiple sclerosis: a consensus statement. Brain. (2024) 147:2913–33. doi:top 10.1093/brain/awae013 38226694 PMC11370808

[71] Calvi A Carrasco FP Tur C Chard DT Stutters J De Angelis F . Association of slowly expanding lesions on MRI with disability in people with secondary progressive multiple sclerosis. Neurology. (2022) 98:e1783–e93. doi:top 10.1212/wnl.0000000000200144 35277438

[72] Rocca MA Preziosa P Filippi M . Progressive multiple sclerosis: Six trials to watch. Med. (2026) 7:100960. doi:top 10.1016/j.medj.2025.100960 41519116

